# Kinesins in *Caenorhabditis elegans* neuronal morphogenesis

**DOI:** 10.1098/rsob.250072

**Published:** 2025-11-26

**Authors:** Shinsuke Niwa, Kyoko Chiba

**Affiliations:** ^1^ Tohoku University, Sendai, Miyagi Prefecture, Japan

**Keywords:** biology, kinesin, *Caenorhabditis elegans*, axonal transport

## Abstract

Neuronal morphogenesis is regulated by intracellular transport and cytoskeletal dynamics. Kinesin superfamily proteins (KIFs), or kinesins, function as molecular motors for intracellular transport and as regulators of the microtubule cytoskeleton, making them essential for neuronal development. *Caenorhabditis elegans* has been widely used as a model organism to study neuronal morphogenesis. Due to the critical roles of kinesins in neuronal functions, numerous kinesin mutants, including unique gain-of-function mutants and temperature-sensitive mutants, have been identified through forward genetic screens in *C. elegans*. The availability of whole-genome knockout resources and CRISPR/Cas9 genome editing has further enabled precise genetic analysis, facilitating the modelling of human kinesin-related diseases in *C. elegans*. In this review, we discuss the functions of *C. elegans* kinesins in neuronal morphogenesis, focusing on their roles in neuronal transport and cytoskeletal regulations.

**Figure:**
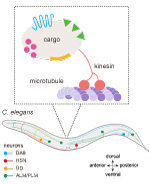

## 1. Introduction

Neurons are specialized cells that have differentiated morphology optimized for transmission and reception of information [1]. To support neuronal morphology, the microtubule cytoskeleton is highly organized, with the plus-end of the microtubules oriented towards the tip of axons. In contrast, dendritic microtubules exhibit a mixed polarity, with both plus- and minus-end-out orientations. In certain invertebrate neurons, such as those of *Caenorhabditis elegans* and *Drosophila*, dendrites can be predominantly minus-end-out, with microtubule minus-ends oriented towards the distal tip. Acting as intracellular transport rails, microtubules enable the delivery of lipids and proteins, which are primarily synthesized in the cell body [1].

Kinesin (now known as Kinesin-1), originally purified from squid neurons and bovine brains in 1985, was identified as a candidate motor for axonal transport [2]. In *in vitro* reconstitution assays, purified kinesin-1 transports cargo organelles, providing strong evidence that it functions as an axonal transport motor. *Caenorhabditis elegans* has been established as a model organism to study neuronal development and functions [3,4]. In the early 1990s, forward genetic screens for body movement and behaviour in *C. elegans* led to the identification of the kinesin-like protein UNC-104 and the kinesin-1 orthologue UNC-116 [5–7] . These findings highlight the physiological roles of kinesins in neuronal function *in vivo*. Notably, in *unc-104* mutant worms, synaptic vesicles mislocalized to the cell body, strongly suggesting that kinesins are essential for anterograde axonal transport [5].

The early history of kinesin research highlights the power of *C. elegans* as a model organism for studying kinesin functions in neurons. EMS mutagenesis has led to the isolation of various unique kinesin mutants, including gain-of-function alleles and temperature-sensitive alleles, providing important mechanistic insights into the regulation and function of kinesins [8,9]. The *C. elegans* research community has conducted whole-genome knockout projects, enabling the use of loss-of-function mutant worms to investigate kinesin function and regulation [10]. With the introduction of CRISPR/Cas9 technology in *C. elegans* in 2013 [11], researchers can now introduce specific mutations into *C. elegans*. This approach has facilitated the generation of disease-model worms, providing insights into the molecular mechanisms underlying kinesin-associated diseases [12–16]. Split GFP technologies enable the labelling of endogenous proteins and organelles without overexpression [17,18]. These findings further underscore the advantages of *C. elegans* as a model system for studying the function of kinesins in neurons.

**Figure 1.**
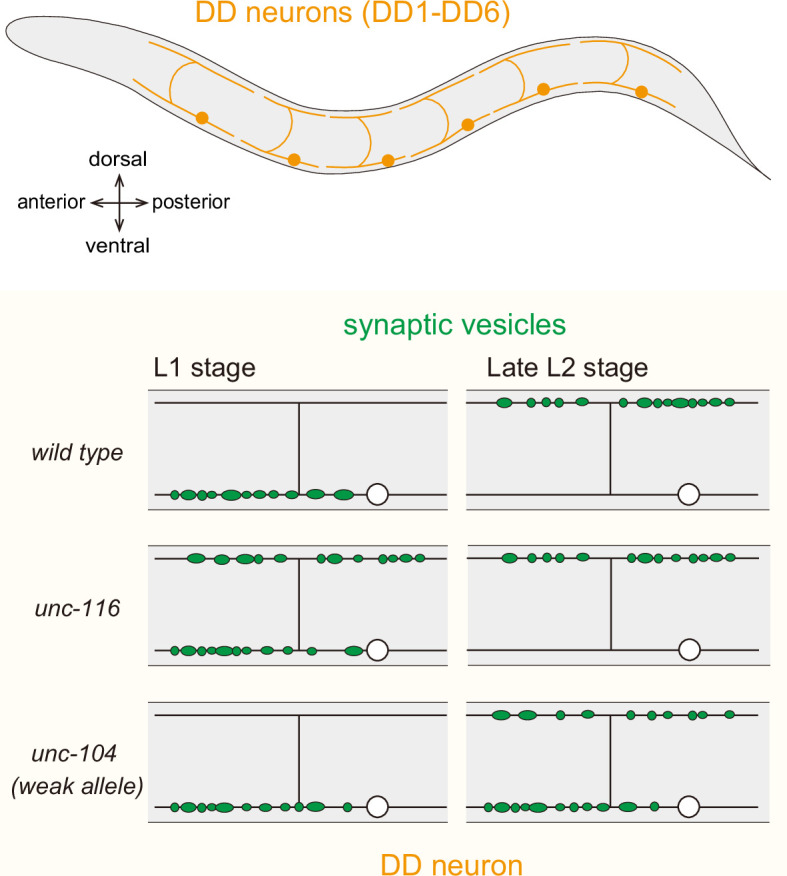
Schematic illustration of DD neurons, which serve as a model for analysing the synaptic remodelling during development. In the L1 stage, synaptic vesicles normally accumulate along ventral neurites and are subsequently relocated to dorsal neurites by the late L2 stage. In *unc-116* mutants, synaptic vesicles abnormally accumulate along dorsal neurites. In weak *unc-104* alleles, synaptic remodelling is delayed, and misaccumulation of synaptic vesicle markers is observed even in the late L2 stage.

**Figure 2.**
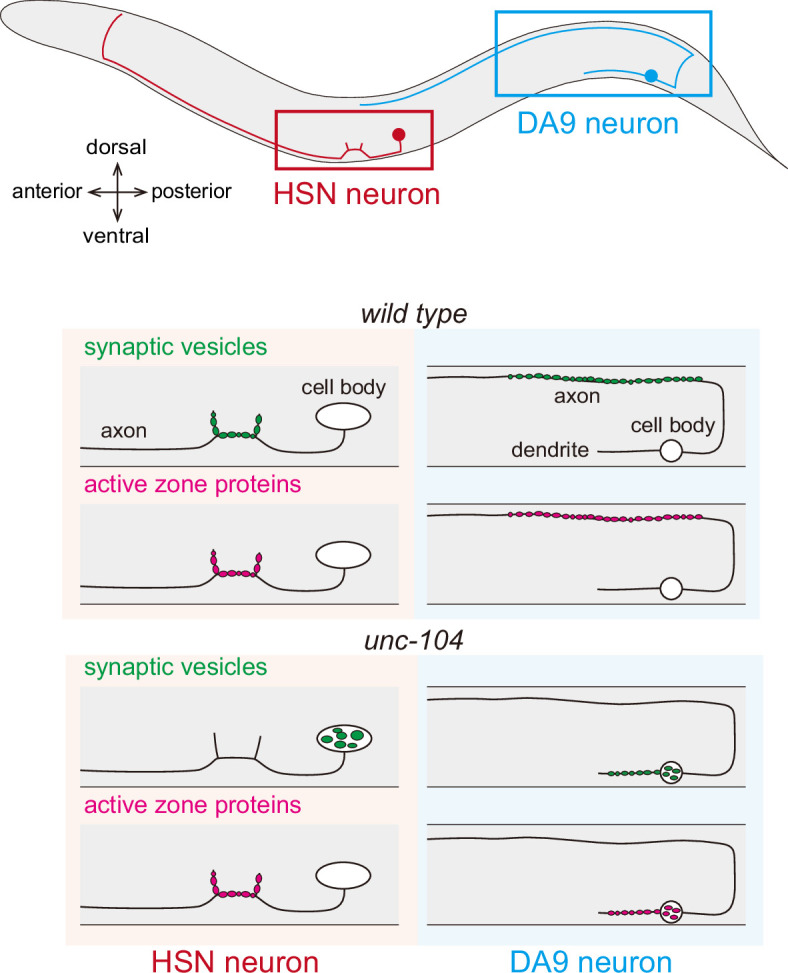
A schematic illustration of DA9 and HSN neurons, which are used to study axonal transport of synaptic components. Both neurons normally form *en passant* synapses along the axon. In *unc-104* mutant HSN neurons, synaptic vesicles abnormally accumulate in the cell body, whereas active zone proteins are correctly transported to synapses. In contrast, *unc-104* mutant DA9 neurons exhibit mislocalization of both synaptic vesicles and active zone proteins along the dendrite and in the cell body.

**Figure 3.**
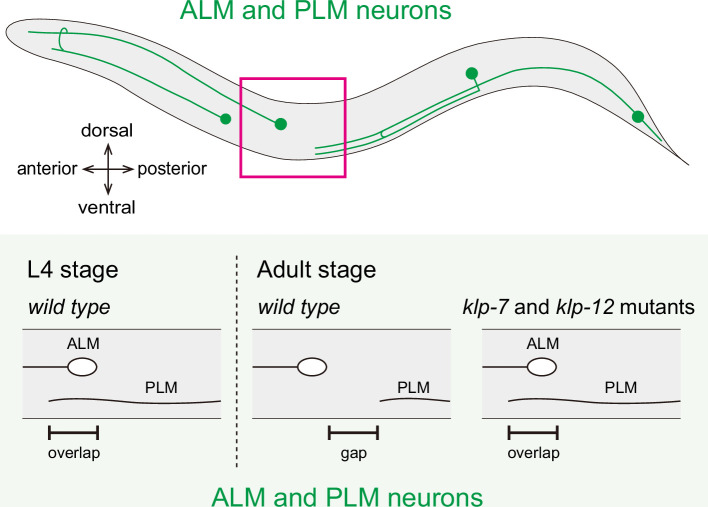
Phenotypes of mechanosensory neurons in microtubule-regulating kinesin mutants A schematic illustration of mechanosensory neurons, which are often studied to analyse the relationship between microtubule dynamics and neuronal morphology. In the L4 stage, the tip of the PLM axon overlaps with the ALM neuron cell body. However, in adult animals, this overlap is typically absent. In *klp-7* and *klp-12* mutant worms, the PLM axon extends beyond its normal length and overlaps with the ALM cell body.

In this review, we discuss the function of *C. elegans* kinesins by focusing on their roles in neuronal morphogenesis (table 1), while many kinesins are involved in intracellular transport in non-neuronal cells, as well as meiosis and mitosis [43]. Numerous review articles have already illustrated how kinesins bind to cargos through adaptor proteins [43,44]. To avoid redundancy, we have omitted such figures. One of the obstacles that hinder researchers from understanding *C. elegans* neurobiology studies is the difficulty of recognizing the localization of *en passant* synapses and the axonal morphology of each neuron. To address this, we have included illustrations showing synaptic localization and axonal morphologies of widely used model neurons and relevant mutant phenotypes (figures 1–3).

**Table 1.** Summary of kinesins and their functions described in this review.

	human orthologue	function	mutant phenotype
**kinesin-1**			
heavy chain: UNC-116	KIFSA, KIF5B, KIF5C	intracellular transport	mislocalization of synapses [19,20] mislocalization of mitochondria [21–24] mislocalization of MTOCs [25,26] mislocalization of glutamate receptor [27] (hypomorphic allele) reduced dense core vesicles [28] (auxin degron alleles)
light chain: KLC-1	KLC1, KLC2, KLC3, KLC4	intracellular transport	mislocalization of mitochondria [24] (null allele)
light chain: KLC-2	KLC1, KLC2, KLC3, KLC4	intracellular transport	mislocalization of synapses [19,20] (null allele)
**kinesin-2**			
KLP-20/KLP-11/KAP-1	KIF3A/KIF3B (or KIF3C), KAP-3	intraflagellar transport	cillia defects [29,30] (null and hypomorphic alleles)
KLP-20	KIF3A	intracellular transport?	abnormal epidermis [31] (null and hypomorphic alleles)
OSM-3	KIF17	intraflagellar transport	cilla defects [29,30] (null and hypomorphic alleles)
**kinesin-3**			
UNC-104	KIF1A, KIF188	intracellular transport	mislocalization of synapses [5] (null and hypomorphic alleles)
KLP-4	KIF13A	intracellular transport	mislocalization of glutamate receptor [27,32] (null and hypomorphic alleles)
KLP-6	KIF28P is classified as pseudogene	intraflagellar transport	mislocalization of PKD-2/LOV-1 receptor male mating defects [33] (null allele)
**kinesin-4**			
KLP-19	KIF4A	inhibition of microtubule polymerization?	lethal [34,35] (null allele)
KLP-12	KIF21A, KIF21B	inhibition of microtubule polymerization	longer axon [36] (null and hypomorphic alleles)
**kinesin-8**			
KLP-13	KIF19 (KIF18A? and KIF18B7)	microtubule depolymerization?	longer oilia [37] (null allele)
**kinesin-11**			
VAB-8	KIF26A? and KIF26B?	regulation of cell migration regulation of axonal transport	cell migration defects [38,39] (null and hypomorphic alleles) mislocalization of synapses [40] (null allele)
**kinesin-13**			
KLP-7	KIF2A, KIF2B, KIF2C	microtubule depolymerization?	longer axon [41] (null allele) cell polarity defects [42] (null allele)

## 2. Kinesin-1 (UNC-116, KLC-1 and KLC-2)

Kinesin-1, widely known as kinesin, is a founding member of the kinesin superfamily proteins [2]. Kinesin-1 is a heterotetramer composed of two subunits of kinesin heavy chains and two subunits of kinesin light chains. Kinesin heavy chain is encoded by the *unc-116* gene in *C. elegans* whereas mammals have three kinesin heavy chain genes, *KIF5A*, *KIF5B* and *KIF5C,* which have slightly different biochemical properties [7,45]. Mutations in *KIF5A*, *KIF5B* and *KIF5C* are causes of neurological disorders such as amyotrophic lateral sclerosis (ALS) [46–50]. UNC-116 has a motor activity *in vitro* [51,52]. Two kinesin light chain genes, *klc-1* and *klc-2*, are identified in *C. elegans*, whereas mammals have four KLC genes, *KLC1*, *KLC2*, *KLC3* and *KLC4* [53]. It is noteworthy that there is no correspondence between *C. elegans* and mammalian gene names. It remains unclear whether KLC-1 and KLC-2 have redundant or distinct roles. Although *klc-2* null mutants are lethal, *klc-1* null mutants are viable [19]. This observation suggests a functional distinction between KLC-1 and KLC-2. However, it is also possible that the two proteins are functionally redundant but differ in their spatial or temporal expression patterns. *unc-116* mutant worms were identified through a genetic screening that searches for mutants exhibiting abnormal backward locomotion upon the head touch [7]. The behavioural phenotype indicates the involvement of UNC-116 in neuronal functions. Consistently, several cargos in neurons, such as synaptic materials, dense core vesicles, mitochondria and microtubule organizing centres (MTOCs), are affected in loss of *unc-116* mutant worms [19–22,25,26,28]. Although outside the main focus of this review, *C. elegans* kinesin-1 is also involved in the transport of phagolysosomes in embryos, the translocation of meiotic spindle to the oocyte cortex and nuclear migration [52,54–56]. Because null alleles of *unc-116* are lethal, most studies have analysed hypomorphic alleles or auxin-induced degron systems to study its function.

### 2.1. Kinesin-1 in synaptic vesicle transport

For synaptic vesicle transport, UNC-116, in complex with KLC-2, binds to UNC-16 (JIP3 orthologue) [20]. UNC-16, in turn, binds to UNC-14 [19]. These four factors are essential for proper synaptic vesicle localization in the GABAergic Dorsal D (DD) motor neurons at the L1 stage (figure 1). To understand these studies, one need to understand the morphology of DD neurons. DD neurons undergo synaptic remodelling between the L1 and late L2 stages (figure 1). Initially, at the L1 stage, DD neurons form neuromuscular synapses along their ventral neurites and receive inputs from dorsally projecting cholinergic neurons (figure 1, left panels). By adulthood, they instead receive inputs from ventral cholinergic neurons and form neuromuscular synapses with dorsal body wall muscles (figure 1, right panels). This remodelling, involving both synapse elimination and formation, is completed by late L2 stage. In *unc-116*, *unc-16* and *unc-14* mutants, synaptic vesicles in L1-stage DD neurons are largely mislocalized to dorsal, instead of ventral neurite (figure 1) [19,20], suggesting that these proteins are essential for proper localization of synaptic vesicles.

### 2.2. Kinesin-1 in mitochondrial transport

Another cargo transported by UNC-116 is mitochondria [22–24]. In *Drosophila* and mammals, mitochondrial transport depends on an adaptor complex composed of the mitochondrial outer-membrane Rho GTPase Miro and its binding partner Milton (also known as TRAK, OIP106 and GRIF-1) [57–60]. The Miro-TRAK complex is crucial for linking mitochondria to both kinesin heavy chain and dynein [59]. Notably, kinesin heavy chains directly bind to TRAK1, and kinesin light chain is not required for the anterograde transport of mitochondria [58]. Miro is anchored to the mitochondrial membrane via its C-terminal transmembrane domain and contains two Rho-homologous GTPase domains.

Interestingly, several studies have suggested that the mechanism of mitochondrial transport in *C. elegans* is different from other model organisms, although core factors are conserved. Milton and Miro are encoded by *trak-1* and *miro-1* genes, respectively [24]. Unlike in *Drosophila* and mammals in which mitochondrial transport does not require kinesin light chains, *klc-1* is essential in *C. elegans*. Additionally, mitochondrial transport in *C. elegans* requires *mtx-1* and *mtx-2* genes, which encode metaxins. MTX-1 and MTX-2 interact with MIRO-1 and KLC-1, forming a complex that facilitates UNC-116-dependent transport. Furthermore, while TRAK1 mediates both kinesin- and dynein-dependent mitochondrial transport in other species, *trak-1* in *C. elegans* is required for dynein-mediated transport but is dispensable for kinesin-dependent transport [24]. Another protein, RIC-7, functions with MTX-2 and MIRO-1 to mediate UNC-116-dependent mitochondrial transport [21]. In *ric-7* mutants, mitochondria do not exit from cell bodies [23]. It is noteworthy that RIC-7 was initially identified as a factor essential for neuropeptide secretion [61], suggesting that mitochondria may play a role in this process. Although the interaction between mitochondrial transport and fission machineries remains poorly understood, reduced mitochondrial motility has been observed in loss-of-function mutants of mitochondrial fission regulators such as *fzo-1* (mitofusin orthologue) and *slc-25A46* (UGO1 and SLC25A46 orthologue) [62].

Homology searches based on the amino acid sequence of RIC-7 have not identified homologues in mammals or *Drosophila*. However, a recently developed structure-based search methods may reveal previously unrecognized homologues [63]. In mammals, TRAK1 has been shown to directly activate the autoinhibited state of KIF5 (UNC-116 orthologue, kinesin heavy chain) in *in vitro* reconstitution assays using total internal reflection fluorescence microscopy (TIRF-M) [60]. To clarify the functional relationships among RIC-7, MTX1/2, TRAK-1 and kinesin-1 (UNC-116/KLC-1), and to elucidate mechanistic differences in mitochondrial transport between mammals and *C. elegans*, biochemical reconstitution assays using TIRF-M will be essential.

### 2.3. Kinesin-1 in slow transport

Cytosolic proteins and cytoskeletal proteins are transported by slow axonal transport [64]. This process is mediated by kinesin-1 and kinesin-2 [65–70]. Although the precise mechanism of slow axonal transport remains unclear, a recent study has established *C. elegans* as a valuable model to study slow axonal transport [71]. Using DA9 neuron as a model (figure 2), the slow axonal transport of spectrin, a representative cargo of slow axonal transport [64], has been visualized. Spectrin exhibits both fast and slow movement along the axon, both of which depend on UNC-116, UNC-76 (FEZ1 orthologue) and UNC-69 (SCOC orthologue). UNC-76/UNC-69 complex serves as an adaptor for slow axonal transport of spectrin [71].

It remains largely elusive whether other components of slow axonal, such as synapsin, metabolic enzymes, microtubules and clathrin complex, rely on the same transport mechanism. Moreover, whether the mechanism identified in *C. elegans* is conserved in other species has not been clarified. Although some factors, including HSP70, have been implicated in slow axonal transport [65], the relationship between UNC-76/UNC-69 and these adaptor proteins remains unknown. Genetic analysis in *C. elegans* may help address these questions.

### 2.4. Kinesin-1 in MTOC transport

UNC-116 transports non-centrosomal MTOCs in dendrites, specifically RAB-11-positive endosomes. The microtubule nucleation complex γ-TuRC is associated with RAB-11-positive endosomes and functions as a non-centrosomal MTOC [25]. In wild-type DA9 and PVD neurons, dendritic growth is supported by minus-end-out microtubules along the dendrites [25,26]. However, in *unc-116* mutants, dendrites predominantly contain plus-end-out microtubules. This defect arises because UNC-116 normally transports RAB-11-positive endosomes towards the tip of the dendritic growth cone [25].

In mammalian dendrites, microtubule polarity is mixed. It is not known whether RAB-11-positive endosomes transported by kinesin-1, as in *C. elegans*, can function as MTOCs.

## 3. Kinesin-2 (KLP20/KLP-11/KAP-1 complex and OSM-3)

Kinesin-2 is required for the morphogenesis of cilia and flagellar [72]. In *C. elegans*, cilia form at the tip of neuronal dendrites and serve as sensors of external environment [4]. Unlike other animals, *C. elegans* sperm do not contain motile flagella [73]. Therefore, the main functions of kinesin-2 in *C. elegans* are the neuronal cilia morphogenesis [72]. Two types of kinesin-2 have been identified, heterotrimeric kinesin-2 and homodimeric kinesin-2 [29,72]. In humans, loss-of-function mutations in kinesin-2 genes and intraflagellar transport (IFT) complex genes, which encode cargo transported by kinesin-2, are associated with ciliopathies such as Bardet-Biedl syndrome, Meckel syndrome and Joubert syndrome [74,75]. *C. elegans* serves as a valuable model organism for studying the molecular pathogenesis of ciliopathies [37,76–85].

### 3.1. Heterotrimeric kinesin-2

Heterotrimeric kinesin-2 was first purified from sea urchin embryo using anti-kinesin antibodies [86,87] and was later identified in other organisms [43]. In mammals, heterotrimeric Kinesin-2 consists of KIF3A, KIF3B or KIF3C, and KAP3. KIF3A and KIF3B/C contain the kinesin motor domain, while KAP3 is an accessory subunit with a cargo-binding armadillo repeats domain [43]. In *C. elegans*, KLP-20, KLP-11 and KAP-1 are the, respectively, orthologues of mammalian KIF3A, KIF3B/C and KAP3 [29,30]. Heterotrimeric kinesin-2 transports IFT particles in the initial segment of cilia [29]. In loss-of-function mutant of *klp-20*, *klp-11* and *kap-1*, cilia formation is strongly inhibited. Moreover, a mutation in the variable abnormality-6 (*vab-6*) mutant, which causes a bumpy epidermis phenotype, has been mapped to the *klp-20* gene, whereas neither *klp-11* nor *kap-1* mutants exhibit this phenotype [31]. The *klp-20* mutation in *vab-6* is a loss-of-function mutation. While the phenotype is not neuronal and out of main focus of this review, the observation suggests that *klp-20*, *klp-11* and *kap-1* may have distinct roles, independent of their cilia functions in the heterotrimeric kinesin-2 motor complex.

Consistent with phenotypes observed in *C. elegans*, heterotrimeric kinesin-2 is required for the cilia and flagellar formation in mammals [43,72]. Heterotrimeric kinesin-2 is also involved in the transport of N-cadherin vesicles in neuroepithelium cells and the axonal transport of fodrin-associated vesicles [88,89]. It is also involved in the slow axonal transport of misfolded SOD1 and choline acetyltransferase [69,70]. Whether *C. elegans* heterotrimeric kinesin-2 participates in these transport phenomena remains unrevealed.

### 3.2. Homodimeric kinesin-2

Homodimeric kinesin-2 consists of two subunits of OSM-3, which have a functional motor domain [29,90,91]. In loss-of-function *osm-3* mutant worms, the distal part of cilia is diminished. While heterotrimeric kinesin-2 works in the proximal segment of the cilia, OSM-3 exhibits its activity to the distal segment, facilitating IFT to the ciliary tip [29]. The activity of OSM-3 is tightly regulated by autoinhibition [91]. OSM-3 is directly associated with IFT-B complex via DYF-1 (IFT70) subunit [92]. OSM-3 is the *C. elegans* orthologue of mammalian KIF17, which transports NMDA receptors in neuronal dendrites in mice [93]. Although KIF17 has been implicated in ciliary function [94], it remains unclear whether vertebrate KIF17 plays a role in distal segment of cilia, similar to that of worm OSM-3. KIF17 localizes along the rod outer segment axoneme in mice and *Xenopus* [94]. In *kif17* homozygous mutant zebrafish, subtle morphological defects of olfactory cilia as well as developmental delays in the rod outer segment have been observed [95,96].

## 4. Kinesin-3 (UNC-104, KLP-4, KLP-6)

Three *C. elegans* kinesins, UNC-104, KLP-4 and KLP-6, belong to kinesin-3 family. Unlike kinesin-1 and kinesin-2, kinesin-3 transports cargos as homodimers, and no accessory subunits have been identified [43,97]. Kinesin-3 is characterized by a conserved forkhead-associated (FHA) domain, which is involved in autoinhibition [98–100].

### 4.1. UNC-104 in synaptic vesicle transport

Among *C. elegans* kinesins, UNC-104 is the most well-characterized both genetically and biochemically. Loss of *unc-104* mutant worms was first identified through genetic screens for uncoordinated locomotion [5,6]. Both null and hypomorphic alleles have been isolated. Purified UNC-104 protein has been extensively studied as a model for understanding the activation mechanism of monomeric kinesin-3 motors [101–103]. Mammals have two UNC-104 orthologues, KIF1A and KIF1Bß [13,14,104,105].

UNC-104 is essential for the axonal transport of synaptic cargos, including synaptic vesicles, their precursors, active zone proteins and dense core vesicles [5,97]. In contrast, mitochondrial localization in neurons is unaffected in neurons in *unc-104* mutants [5,23], while a study has suggested UNC-104 transports mitochondria in non-neuronal tail spike cells [106].

Interestingly, depending on the neuronal types, the contribution of *unc-104* in the transport of pre-synaptic cargos is different (figure 2). In DA9 and DD neurons, the localization of both synaptic vesicles, such as RAB-3, and active zone proteins, including UNC-10 (RIM orthologue), CLA-1 (Piccolo and RIM orthologue) and NRX-1 (neurexin orthologue), depend on UNC-104 (figures 1 and 2, right panels) [8,107–109]. Therefore, the location of neuromuscular junctions between these neurons and muscles is regulated by UNC-104 activity. In contrast, the localization of synaptic vesicles is strongly affected in HSN neurons in *unc-104* loss-of-function alleles (figure 2, left panels, green), whereas the distribution of active zone proteins remains largely unaffected (figure 2, left panels, magenta) [110]. Similar synaptic vesicle-specific defects have been reported in other neurons [5]. The molecular mechanism underlying these differences remains unclear. Moreover, the synaptic remodelling of DD neurons depends on the activity of UNC-104 motor [111]. While the synaptic remodelling is normally completed by late L2 stage, the timing is significantly delayed in *unc-104* hypomorphic alleles (figure 1) [111].

The synaptic phenotypes of *unc-116* and *unc-104* loss-of-function mutants in DD neurons are clearly distinct (figure 1). Given that synaptic components are synthesized in the soma and transported along the axon, the phenotype observed in *unc-104* loss-of-function mutants is consistent with impaired anterograde transport. In contrast, the phenotype of *unc-116* loss-of-function mutants is unlikely to result from simple defective axonal transport. The mechanistic relationship between UNC-104 and UNC-116 in synaptic development and maintenance remains to be elucidated. In the hippocampal neuron of *KIF1A* or *KIF1B* knockout mice, synapse numbers are reduced due to the defects in axonal transport of synaptic vesicle precursors [105,112,113], which is similar to the morphology of *unc-104* mutant worms. A similar reduction in synapse number is observed in *Drosophila Imac* mutants, which have mutations in the orthologue of *unc-104* gene [114]. These phenotypes suggest that the function of these motor proteins is evolutionarily conserved. Supporting this, expression of human KIF1A or KIB1Bβ using the *unc-104* promoter can rescue the body movement defects of *C. elegans unc-104* mutants, indicating that the functions of KIF1A, KIF1Bβ and UNC-104 are conserved [13,14]. In *C. elegans*, the localization of active zone proteins depends on *unc-104* in some neurons but not in others, indicating that neuron-type-specific mechanisms exist (figure 2). In mammals, it has been technically challenging to comprehensively study synapses across the entire nervous system. As a result, it remains unknown whether neuron-type-specific differences, such as those observed in *C. elegans unc-104* mutants, also exist.

### 4.2. Regulation of UNC-104

Worm genetics has identified regulatory factors for UNC-104-dependent axonal transport of synaptic cargos, including a small GTPase ARL-8 (ARL8A/ARL8B orthologue) and the BLOC-1-related complex (BORC) [8,108,115–117]. In loss-of-function mutants of *arl-8* and BORC subunits such as *sam-4*, synaptic vesicles are mislocalized to the cell body and proximal segment of the axon in DA9 neuron and PLM neurons. Time-lapse observation shows that axonal transport of synaptic proteins, such as RAB-3 and UNC-10, is reduced [116,117]. Genetic analysis suggests that BORC functions upstream of ARL-8, which in turn up-regulates UNC-104 [116]. Moreover, biochemical studies have shown that an active zone protein SYD-2 directly binds to UNC-104 [118]. Unlike *unc-104*, *arl-8* and BORC mutants, where synaptic vesicles accumulate in the proximal axon and cell body, *syd-2* mutants exhibit synaptic vesicle mislocalization across all neuronal compartments in DA9 [108]. While *arl-8* loss-of-function mutations reduce axonal transport, *syd-2* loss-of-function mutations serve as suppressors for *arl-8* mutants by increasing the amount of axonal transport. These suggest that the role of *syd-2* in axonal transport is different from those of *arl-8* and BORC [108].

At the synapses, UNC-104 dissociates from synaptic vesicle precursors and is subsequently degraded. This degradation of UNC-104 requires the E1 ubiquitin-activating enzyme UBA-1 [119]. Furthermore, an unidentified post-translational modification, which is mediated by a F-box protein FBXB-65, is required for UNC-104 degradation. In *fbxb-65* knockdown worms, UNC-104 abnormally misaccumulates at neuronal ends [120].

In mammals, a study suggests that BORC and ARL8B are not required for the axonal transport of synaptic vesicle precursors [121]. While BORC and ARL8B are required for the transport of lysosomes, their contribution in worm neurons is not clear as lysosomes are not transported to axons, at least in the DA9 [108]. These raise the question of whether the BORC-ARL-8-UNC-104 pathway is evolutionarily conserved. Conversely, expression of mammalian ARL8 can rescue the phenotypes of *arl-8* mutant worms, strongly suggesting that this transport mechanism is conserved between mammals and *C. elegans*.

### 4.3. UNC-104 and disease model worms

Both gain-of-function and loss-of-function mutants of *unc-104* have been used to study the molecular mechanisms of KIF1A-associated neurological disorder (KAND) and Charcot-Marie-Tooth disease type 2A (CMT2A) [13–15]. Because the expression of human KIF1A and KIF1Bß using the *unc-104* promoter rescues the motility defects of *unc-104* loss-of-function mutant worms, this system allows researchers to test whether human *KIF1A* and *KIF1Bß* variants are loss-of-function mutations [13,14]. Furthermore, using CRISPR/Cas9, several disease model worms for KAND and CMT2A have been established. By using these disease models, studies have revealed that KAND is caused by both increased axonal transport and reduced axonal transport [12,13], whereas CMT2A is caused by impaired axonal transport [14]. Interestingly, the *unc-104(R9Q*) mutant, a model for KAND, can be rescued by treatment with a plant flavonol fisetin [15]. These findings suggest that *C. elegans* disease models may be useful for identifying novel remedies to treat genetic disorders.

### 4.4. KLP-4 in neuronal receptor transport

KLP-4, the *C. elegans* orthologue of mammalian KIF13A, was identified in a genetic suppressor screen of *cdk-5-*overexpressing mutants using the glutamate receptor GLR-1 as a marker [122]. In *C elegans* ventral nerve cord (VNC), CDK-5 is essential for the transport of GLR-1 [122]. In loss of *cdk-5* mutant worms, the amount of GLR-1 decreases along the VNC. Inversely, *cdk-5*-overexpressed VNC exhibited increased amount of GLR-1. A suppressor screen for *cdk-5*-overexpressing mutant worms with elevated dendritic GLR-1 identified a loss-of-function allele of *klp-4*. In *klp-4* null mutants, the amount of GLR-1 is reduced in VNC [32]. In a subsequent study, it has been observed that another kinesin UNC-116 is also required for the GLR-1 transport. It has been suggested that UNC-116 is required for the long-range transport in the dendrite, whereas KLP-4 is required for the exporting from the cell body [27].

In mammals, the KLP-4 orthologue KIF13A is required for the dendritic transport of serotonin receptors [123], whereas its role in glutamate receptor transport remains unknown. It would be interesting to study whether *C. elegans klp-4* contributes more broadly to dendritic receptor transport, and whether expression of mammalian KIF13A can rescue the phenotypes of *klp-4* mutants, providing insight into the evolutionary conservation of these kinesins.

### 4.5. KLP-6 in mechanoreceptor transport in cilia

KLP-6 was isolated through a forward genetic screening for mutants exhibiting defects in male mating behaviour [33]. KLP-6 is expressed in male-specific neurons and transports mechanoreceptor complex composed of LOV-1 (PKD1/Polycystin-1 orthologue) and PKD-2 (PKD2/Polycystion-2 orthologue), which localize to male cilia. LOV-1 and PKD-2 are expressed in the male-specific ciliated sensory neurons essential for male mating behaviour [33]. Consistent with the localization of the mechanosensory receptor complex, KLP-6 moves along the axoneme of *C. elegans* cilia [124]. In male cilia, KLP-6 cooperate with heterotrimeric kinesin-2 (KLP-20/KLP-11/KAP-1) and homodimeric kinesin-2 (OSM-3) to transport the mechanoreceptor. Notably, KLP-6 is the first kinesin superfamily protein whose full-length structure was determined [100]. Its biochemical property is unique. The full-length KLP-6 protein moves processively along microtubules as a true monomer *in vitro* [125]. The microtubule-binding tail domain is required for the movement. However, forced dimerization of KLP-6 improves its motility *in vitro*, suggesting that KLP-6 may form dimers through an unidentified mechanism [103,125].

Similar to worms, the mechanosensory receptor complex composed of Polycystin-1 and Polycystin-2 is localized in the cilia in mammalian kidney tubular epithelial cells and biliary duct cholangiocytes [126]. They are believed to regulate renal tubule and bile-duct diameter via sensing unidentified stimuli [127]. However, while human genome encodes a KLP-6 orthologue sequence, *KIF28P*, the gene is classified as a pseudogene. Why KLP-6 is not required for the mechanoreceptor transport in mammalian cilia remains to be elusive. One possibility is another kinesin can compensate the function of KLP-6 in mammalian cilia.

## 5. Microtubule-regulating kinesins (KLP-7, 12, 13 and 19)

Motor domains from several kinesins, kinesin-4, kinesin-8 and kinesin-13 are structurally adapted to regulate microtubule dynamics [36,128–132]. Kinesin-8 and kinesin-13 are well established as microtubule-depolymerizing kinesins. *C. elegans* has one Kinesin-8 and one Kinesin-13 gene, respectively, named KLP-13 and KLP-7 [37].

### 5.1. KLP-13 in ciliary length control

In mammals, kinesin-8 regulates spindle length and ciliary length [133–135]. Amino acid sequence suggests *C. elegans* KLP-13 is an orthologue of mammalian KIF19 [136]. Similar to KIF19, KLP-13 accumulates at the tip of cilia [37]. Slightly longer cilia in ADL neuron, less than 10% longer in length, are observed in *klp-13* mutant worms. The longer cilia phenotype is similar to KIF19-knockout mice exhibiting longer cilia in ciliated epithelial cells of the oviduct and brain ventricles. The severity of this phenotype varies between tissues in mice [136] (and our unpublished observations), raising a possibility that cilia in other neurons may be more strongly affected in *klp-13* mutants.

### 5.2. KLP-7 in neurite length control

KLP-7 is the solo orthologue of mammalian KIF2A, KIF2B and KIF2C [42]. KIF2A, KIF2B and KIF2C depolymerize microtubules and regulate spindle formation [137]. KIF2A is expressed in neurons, localizing to the growth cone and regulating microtubule dynamics [138,139]. KLP-7 performs the function of KIF2A, KIF2B and KIF2C and regulates both spindle length and neuronal microtubules in *C. elegans* [41,140]. To study the function of KLP-7, PLM and ALM neurons have been analysed [41] (figure 3). The neurodevelopmental properties of ALM and PLM mechanosensory neurons are suitable for studying neurite length. At the late L4 stage, the PLM axon tip typically overlaps with the ALM cell body. By the adult stage, however, the PLM neurite retracts, creating a gap between the PLM axon tip and the ALM cell body (figure 3). *klp-7* null mutants exhibit longer axons in PLM neurons (figure 3) [41]. Interestingly, loss of *klp-7* caused a mislocalization of axonal proteins, including RAB-3, SAD-1, and their motor UNC-104, to dendrites in PVD neuron [42]. UNC-44 (ankyrin orthologue), a conserved axon initial segment (AIS) protein, is essential for the compartmentalization of axonal proteins in *C. elegans* [141]. In wild-type PVD neurons, a 6994 aa isoform of UNC-44 localizes to the AIS to maintain neuronal polarity [141,142]. However, in *klp-7* null mutants, UNC-44 is mislocalized to the dendrites. Although the precise mechanism remains unclear, regulation of microtubule dynamics by KLP-7 is considered to be necessary for the proper localization of UNC-44. This regulation is fundamental for neuronal polarization and the establishment of axonal and dendritic compartmentalization [42].

In mammals, KIF2A knockout mice exhibit longer axonal branches [138], which is similar to the phenotype of *klp-7* null mutant neurons (figure 3). De novo mutations in KIF2A are associated with cortical dysplasia and microcephaly in human. These suggest that the function of these kinesins is evolutionarily conserved.

## 5.3. KLP-12 in axonal length control

Kinesin-4 family members regulate microtubule length by suppressing microtubule dynamics [36,131,132,143]. *C. elegans* has two kinesin-4 family members, KLP-12 and KLP-19. KLP-19 is an orthologue of chromosome kinesin KIF4A and is involved in chromosome transport during prometaphase [34,35,144], but its role in neuronal morphogenesis remains unclear. KLP-12 is the orthologue of KIF21A and KIF21B [36], all of which have conserved WD40 repeats in their tail domains. To study the function of KLP-12 in axonal length control, ALM and PLM neurons have been analysed (figure 3). Loss-of-function mutations in *klp-12,* both hypomorphic and null alleles, exhibit longer axon phenotypes and axonal guidance defects in ALM and PLM neurons (figure 3). *In vitro*, purified KLP-12 has a plus-end directed motor activity and suppresses microtubule dynamics, suggesting that KLP-12 limits axonal length by regulating microtubule dynamics [36].

Gain-of-function mutations in human *KIF21A* lead to KIF21A overactivation and cause congenital fibrosis of the extraocular muscles 1 (CFEOM1), a disorder characterized by axonal guidance defects [143,145]. These abnormalities arise from the misregulation of axonal microtubule dynamics. Thus, by introducing the corresponding mutations into the *klp-12* gene using CRISPR/Cas9 technology, gain-of-function mutants could be established to generate CFEOM1 model *C. elegans*.

## 6. Non-motile kinesin (VAB-8)

VAB-8 is the *C. elegans* orthologue of mammalian KIF26A and KIF26B, which lack a functional P-loop in their motor domains and are classified as non-motile kinesins [43,146]. Consistently, neither VAB-8 nor KIF26A exhibits motility on microtubules *in vitro* [40,146]. Initially identified as essential for neuronal and growth cone migration, VAB-8 was later implicated in regulating SAX-3 receptor localization at the cell surface [38,39]. Since VAB-8 lacks motor activity, its role in SAX-3 receptor transport remains unclear.

Despite being non-motile, VAB-8 contributes uniquely to axonal transport [40]. Through a genetic screen, VAB-8 was identified as an essential factor for proper presynaptic location in DA9 neuron. Synapses are ectopically localized anteriorly. VAB-8 localizes to microtubule minus ends throughout DA9 neurons. Interestingly, presynaptic sites coincide with minus ends in DA9 neuron. In *vab-8* loss-of-function mutants, RAB-3 vesicles pause less frequently and exhibit longer run lengths, indicating that VAB-8 promotes cargo pausing. Reducing dynein rescues both synapse number and positioning, suggesting that VAB-8 facilitates dynein pausing at minus ends to ensure proper cargo delivery to presynapses [40].

## 7. Perspectives

Additional perspectives not fully discussed in the main sections are briefly mentioned below.

### 7.1. *In vitro* biochemical assays

Genetic studies have demonstrated that various kinesins regulate neuronal morphogenesis. However, biochemical analyses have not been performed in most cases. The presence of a conserved kinesin motor domain does not necessarily indicate motor activity, necessitating direct biochemical characterization. While the microtubule-dependent motor activities of UNC-104 and OSM-3 were established early [91,101], those of UNC-116 and KLP-6 were confirmed recently [51,103]. Similarly, phylogenetic analyses suggest that KLP-13 and KLP-7 function as microtubule depolymerizers [37,147], yet direct biochemical evidence is lacking. Although these fundamental studies may seem trivial, they are essential for using the genetic data available in *C. elegans* to understand kinesin regulation. *C. elegans* genetics has identified many point mutations in kinesin-encoding genes. These genetic insights give valuable insights for reconstituting kinesin activation mechanisms. For instance, by analysing UNC-104 motors carrying point mutations obtained through EMS mutagenesis, monomer-to-dimer transition of UNC-104 can be clearly observed, revealing a link between autoinhibition release and dimerization [103]. Similarly, a point mutation in OSM-3, found by EMS mutagenesis, unlocks the autoinhibition and increases the association with microtubules *in vitro* [91]. Many other kinesin point mutations have been identified, and biochemical characterization of these variants may provide further insight into the regulatory mechanisms of kinesins.

### 7.2. *In vitro* reconstitution assays

To fully understand the regulation and function of motor proteins, *in vitro* reconstitution of transport machineries is essential [2,45,60,148]. As a crucial next step, such reconstitution should focus on complexes identified through *C. elegans* genetics, such as essential components for slow axonal transport (UNC-116/UNC-76/UNC-69) [71], synaptic vesicle transport (UNC-116/KLC-2/UNC-16/UNC-14, UNC-104/BORC/ARL-8 and UNC-104/SYD-2) [19,116,118] and mitochondrial transport (UNC-116/KLC-1/RIC-7/MTX-1/MTX-2/MIRO-1/(TRAK-1?)) [21,24]. Reconstitution of these transport system *in vitro* would provide valuable mechanistic insights into kinesin-mediated transport. As discussed in this review, emerging evidence suggests that distinct transport mechanisms may operate in *C. elegans* and mammals in synaptic vesicle and mitochondrial transport. Reconstitution studies could elucidate the molecular basis of these differences and clarify which elements are conserved and which have diverged across species.

## Data Availability

This article has no additional data.
